# Germline replacement by blastula cell transplantation in the fish medaka

**DOI:** 10.1038/srep29658

**Published:** 2016-07-13

**Authors:** Mingyou Li, Ni Hong, Hongyan Xu, Jianxing Song, Yunhan Hong

**Affiliations:** 1 Ministry of Education Key Laboratory of Exploration and Utilization of Aquatic Genetic Resources, College of Fisheries and Life Sciences, Shanghai Ocean University, 999 Hucheng Huan Road, Shanghai 201306, China; 2 Department of Biological Sciences, National University of Singapore, Science Drive 4, Singapore 117543, Singapore; 3 Institute of Bioengineering and Nanotechnology, 31 Biopolis Way, Singapore 138669, Singapore

## Abstract

Primordial germ cell (PGC) specification early in development establishes the germline for reproduction and reproductive technologies. Germline replacement (GR) is a powerful tool for conservation of valuable or endangered animals. GR is achievable by germ cell transplantation into the PGC migration pathway or gonads. Blastula cell transplantation (BCT) can also lead to the chimeric germline containing PGCs of both donor and host origins. It has remained largely unknown whether BCT is able to achieve GR at a high efficiency. Here we report efficient GR by BCT into blastula embryos in the fish medaka (*Oryzias latipes*). Specifically, *dnd* depletion completely ablated host PGCs and fertility, and *dnd* overexpression remarkably boosted PGCs in donor blastulae. BCT between normal donor and host produced a germline transmission rate of ~4%. This rate was enhanced up to ~30% upon PGC boosting in donors. Most importantly, BCT between PGC-boosted donors and PGC-ablated hosts led to more than 90% fertility restoration and 100% GR. Therefore, BCT features an extremely high efficiency of fertility recovery and GR in medaka. This finding makes medaka an ideal model to analyze genetic and physiological donor-host compatibilities for BCT-mediated surrogate production and propagation of endangered lower vertebrates and biodiversity.

Sex and germline are the basis of reproduction, and their manipulation is essential for reproductive engineering and infertility treatment^1^,^2^,^3^. The germline is established by primordial germ cell (PGC) specification early in developing embryos of diverse animal species^1^,^4^,^5^,^6^. PGCs do not stay in the site of their formation but migrate through a somatic route into the developing gonad^5^. In adult animals, gonadal germ cells undergo meiosis and produce gametes, namely eggs in female and sperm in male^6^,^7^. Germline replacement (GR) is a powerful reproductive technique and holds enormous potential in maintenance and propagation of scientifically or commercially valuable animals and more importantly, in restoration and propagation of endangered species even from cryopreserved germ cells or germline-competent cells for biodiversity conservation. GR is achieved by transplantation of donor cells into hosts, leading to the production of germline chimeras capable of producing donor-derived gametes for germline transmission to next generations. Surrogate reproduction by transplantation between different transplantation donor and host is an emerging paradigm for restoration and propagation of endangered species. In fish, Yoshizaki *et al*. first reported surrogate production of rainbow trout in salmon hosts^2^,^8^.

Germ cell transplantation (GCT) has so far been the gold standard for GR in diverse organisms such as fish. Multiple GCT procedures have been developed with respect to germ cell origin, source and treatment, donor-host combination, developmental stage, place and treatment of host. Yoshizaki and his colleagues have pioneered GCT in salmonids by using normal or triploid embryonic gonads as hosts and reported several important scientific advances and innovative procedures for GR in surrogate production^2^,^8^,^9^,^10^. Procedures have also been established for GCT into busulfan-sterilized host gonads of maturing or matured tilapia^11^ and atherinopsids^12^, for GCT into the embryonic peritoneal cavity as part of the PGC migration pathway in jack mackerel^13^ and tilapia^14^, and into blastula host in medaka^15^. An attractive alternate to GCT is blastula/blastocyst cell transplantation (BCT). The inner cell mass cells of mammalian blastocyst embryos are pluripotent and competent for PGC formation even after transplantation into host blastocysts. Embryonic stem (ES) cells are similar to the ICM and have widely been used for the production of germline chimeras via blastocyst transplantation. In zebrafish, BCT produces germline chimeras at a low efficiency by using normal blastula embryos^16^,^17^ but at a high efficiency in *dnd* depleted blastula embryos^18^. BCT is also able to produce germline chimeras in loach^19^.

The laboratory fish medaka (*Oryzias latipes*) is an excellent lower vertebrate model to analyze and manipulate embryonic development^20^. We make use of this fish for reproductive biology and biotechnologies. Medaka has diploid embryonic stem (ES) cells capable of somatic chimera production, haploid ES cells capable of whole animal production^21^, male germ stem cells capable of test-tube production^7^ and transgenic lines for visualization of PGCs and adult germ cells^22^,^23^. Medaka PGCs from early embryonic cells can be specified in culture^22^ and are able to colonize the host germline upon blastula transplantation^15^. PGC preformation appears to operate in medaka^24^ as in zebrafish^25^. However, germ plasm components in medaka do not localize as in zebrafish^25^,^26^ but rather distribute widely^27^,^28^. The fate and number of PGC precursors are determined by *dnd* whose RNA forms particles and asymmetrically segregates into 8 blastomeres of 32–64 cleavage embryos. Altering the level of *dnd* expression can greatly alter the PGC number, ranging from remarkable increase by nearly 3-folds to complete absence of PGC specification^1^. In addition, medaka is the best studied lower vertebrate for sex determination^29^,^30^,^31^, initiation^32^ and maintenance^33^.

We are developing the BCT as a necessary complementary approach to GCT for GR in basic and applied research. Previously, we and others have established procedures and parameters for the efficient production of somatic and germline chimeras via BCT^34^,^35^,^36^ and ES cell transplantation^21^,^34^,^35^,^37^. Here we report the BCT procedure and parameters for 100% GR. We show that *dnd* depletion is 100% efficient for host sterilization via abolishing PGC formation, and that *dnd* overexpression remarkably enhances the efficiency of germline chimera formation via increasing the PGC number in donor blastula cells. Strikingly, BCT between PGC-boosted donor and PGC-ablated host leads to a 90% high efficiency of fertility restoration and 100% GR. This finding makes medaka an ideal model organism to analyze biological and technical donor-host compatibilities for BCT-mediated surrogate production and propagation of endangered lower vertebrates and their biodiversities.

## Results

### Donor strain and treatment

**Figure 1:**
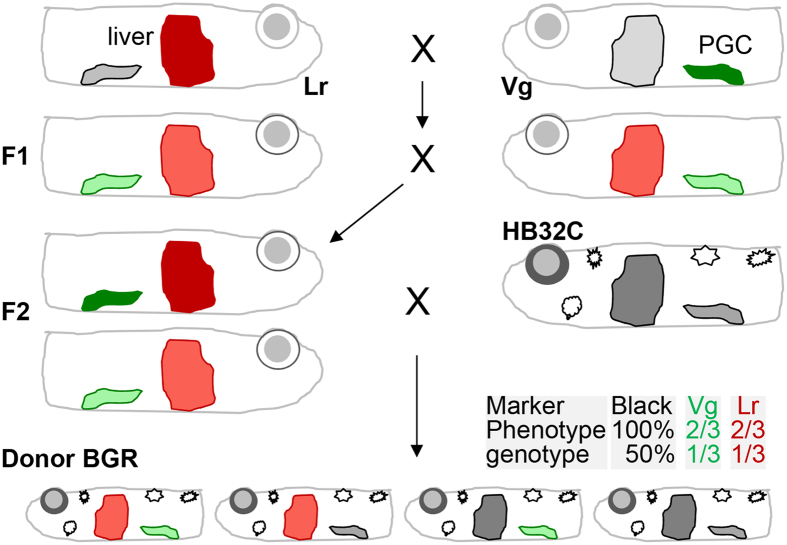
Preparation of donor BGR. Lr and Vg are transgenic *i*^*3*^ albino fishes that express RFP in the liver (Lr for liver red) and GFP in germ cells (Vg). F2 fish positive for both Vg and Lr from the cross between Lr and Vg were mated with wild-type (black) pigmentation strain HB32C. The resultant BGR embryos and fish were all heterozygous for black pigmentation (stars) but only 2/3 positive for either Lr (red) or Vg (green).

The ability to assess the GR efficiency by BCT is the availability of easily visible markers capable of distinguishing donor and host contribution to germline transmission. We generated BGR donor embryos that offer 3 dominant traits, namely wild-type melanocytes for black pigmentation, GFP-labeled germ cells (Vg) and RFP-labeled liver (Lr for liver red). BGR is 100% phenotypically pigmented and genetically heterozygous (50%) for wild-type melanophores, and 66.6% positive for GFP-labeled PGCs or RFP-labeled liver but 33.3% for Vg or Lr transgenes according to the breeding scheme (Fig. 1). Embryo observations confirmed the predicted percent values of the phenotypes (Table 1).

In GCT, the majority if not all of donor germ cells are capable of contributing to the host germline. In BCT, only a minority of donor cells have this capability. In medaka, for example, a blastula embryo has approximately 1000 cells including 33 PGCs^1^,^22^,^38^. The standard BCT procedure transplants approximately 30 blastula cells and thus just one single PGC on average into each blastula host, and the proportional contribution by donor PGCs will be only 3% of the germline of resultant chimeras. In order to enhance the PGC proportion in donor blastula cells, BGR embryos were injected at the 1-cell stage with 100 pg of dnd:ch mRNA and examined for the PGC number at the blastula stage. A non-injected control BGR embryo on average was found to have 33.1 PGCs, and this number was increased by 2.5-folds to 82.5 (Table 1). Notably, the generation of more PGCs by *dnd* RNA injection at the 1-cell stage did not alter the percentage values of phenotypic traits. Thus, the PGC proportion in donor embryos can greatly be increased by *dnd* overexpression.

### Host strain and treatment

We used medaka strain *i*^*3*^ as the BCT host. This strain displays albinism and does not contain any transgene. The host was treated in two ways to favor germline chimera formation. Previously we have shown that γ-irradiation can compromise hosts to increase donor cell contribution into the liver and gonad^34^, and that *dnd* depletion can completely prevent PGC specification^1^. Both approaches were adopted for host treatment. Host embryos were exposed to γ-irradiation at 4–8 cell stages or injected at the 1-cell stage with 20~100 pg of MOdnd, an antisense morpholino oligo capable of specifically inhibiting medaka *dnd* mRNA translation^1^. Control and treated embryos were grown into adulthood and examined for fertility by progeny test. As shown in Table 2, approximately 98.3% (n = 59) of adults from control embryos were fertile; γ-irradiation dramatically reduced the survival rate from 44.7% of control embryos to 9.8% and produced 94.6% fertile adults; and dnd depletion by injecting MOdnd did not affect the survival rate but severely reduced % fertility at 20 pg (32.2%, n = 31) or completely abolished fertility (100%) at 50 pg (n = 49) and 100 pg (n = 98). Thus, γ-irradiation is inefficient for host sterilization, whereas dnd depletion is highly efficient for host sterilization via completely abolishing PGCs.

### Blastula cell transplantation

**Figure 2:**
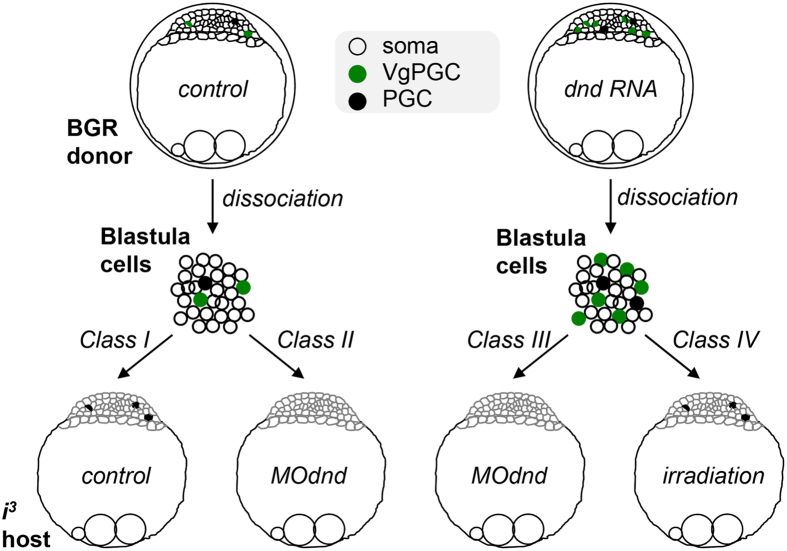
Donor and host treatment and BCT scheme. BGR donor embryos at the 1-cell stage were not injected (control) or injected with 100 pg of dnd:ch RNA (*dnd* RNA) and dissociated at the midblastula stage into single blastula cells. Albino *i*^*3*^ host embryos at the 1-cell stage were not injected (control) or injected with 20–100 pg of dnd-targeting morpholino oligo MOdnd (MOdnd) and dechorionated for BCT at the midblastula stage. Alternatively, Host embryos at the 4–8-cell stages were subjected to gamma-irradiation at 6 gy and used for BCT at the midblastula stage. Approximately 30 blastula cells were transplanted into the deep cells of a blastula host. BCT were performed between normal donor and host (class I), dnd RNA-injected donor and normal (class II), MOdnd-injected (100 pg; class III) and irradiated hosts (class IV). Note that *dnd* RNA increases PGCs in donor blastula cells and that MOdnd abolishes PGCs in the host blastulae.

We performed 4 classes of BCT on the basis of donor-host combinations (Fig. 2). Class I is BCT between normal donor and host, class II between *dnd*-overexpressing donor and normal host, class III between *dnd*-overexpressing donor and *dnd*-depleted host, and class IV between dnd-overexpressing donor and γ-irradiated host. In each class, approximately 30 blastula cells were transplanted into the deep cells of a blastula host. It was found that chimeras of classes I-IV were 100% positive for pigmented melanophores of donor origin (Table 2), demonstrating the successful and reproducible BCT into hosts of an albino background.

We made use of PGCs to precisely evaluate the chimera frequency and chimerism degree (Table 1; Table S1). The former refers to % chimera production, and the latter to the number or proportion of donor-derived cells as a precise measure for relative donor contribution in chimeras. The initial number of PGCs produced by an embryo is well-known in medaka and can easily be counted during somitogenesis when PGCs are positioned bilaterally to somites along the axis. A total of 36 class-I chimeras were examined, 21 and 17 of them were found to be positive for Lr and Vg, respectively, producing chimera frequencies of 58.3% and 47.2%. The 17 Vg-positive chimeras had 1.35 Vg-PGCs on average, thus a chimerism degree of 1.35. When calibrated to 66.7% for phenotypically VgPGCs, the chimera frequency and chimerism degree were predicted to be 63.5% and 1.56 for total PGCs. Since a control blastula embryo produces 33.1 PGCs on average, the 1.56 donor PGCs will have 5% proportional contribution to the PGC pool of class-I chimeras. Therefore, class-I BCT is able to produce a low efficiency of germline chimera production.

*Dnd*-overexpression increased the PGC number per embryos by 2.5 folds from 33.1 in control embryos to 82.5 in the dnd RNA-injected embryo. Transplantation of such PGC-boosted donor blastula cells into control hosts (class-II BCT) significantly increased the chimeras frequency and chimerism degree to 81.2% and 2.04 for Vg, the values become 92.4% and 2.66 upon extrapolation to total PGCs (compare class I and II; Table 2). On the other hand, host treatment by γ-irradiation or dnd depletion did not alter the chimera frequency and chimerism degree (compare class II and III or IV). Notably, pretreatments of donor and host embryos had no significant effect on somatic chimera production. One exception to this was an increased Lr chimera frequency to 75% in γ-irradiated hosts compared with 56.2–58.3% in control and *dnd* RNA-injected hosts (Table 1), which is consistent with our previous observation that γ-irradiation promotes donor ES cell’s contribution to the liver^34^. Taken together, the use of *dnd*-overexpressing donor blastula cells remarkably increases the input number of donor PGCs and thus the germline chimera frequency and chimerism degree without affecting somatic chimera formation, and the use of dnd-depleted hosts does not alter the input number of donor PGCs and thus the formation of germline chimeras without altering somatic chimera formation.

### Donor PGC migration and proliferation

**Figure 3:**
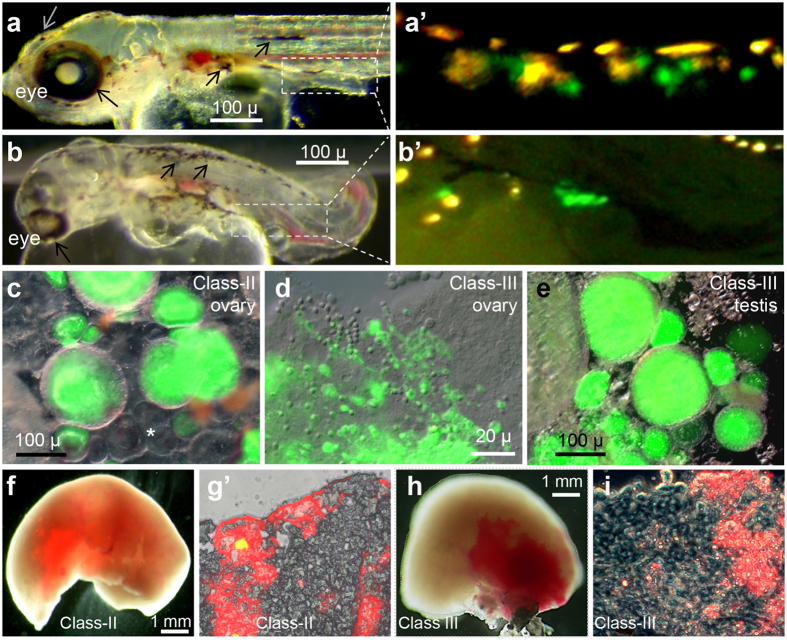
Chimeric fry and adult organs. (**a** and **a**’) Class-III chimera, showing normal development and donor-derived melanocytes (arrows) on the body surface and PGCs (green) in the gonad. **(b** and **b’**) Class-IV chimera, showing developmental defects (bent body, curved tail and abnormal eye) and donor-derived melanocytes (arrows) on the body surface and PGCs (green) in the gonad. (**c)** Flat-mounted ovary of a class-II chimera, showing few GFP-positive oocytes and many GFP-negative oocytes (asterisk). (**d**,**e**) Flat-mounted testis (**d)** and ovary (**e**) of class-III chimeras, showing GFP-positive germ cells (green). (**f**,**g**) Liver of a class-II chimera, showing RFP-positive cells in the liver and its flat-mounted part. (**h**,**i**) Liver of a class-III chimera, showing RFP-positive cells in the liver and its flat-mounted part.

BCT-introduced donor PGCs were able to migrate into the embryonic gonad and increased their number. On average, a chimera of classes II-IV could receive ~2.5 PGCs or ~2 VgPGCs (because only 2/3 of PGCs is Vg-positive). The majority of classes II-IV chimeras indeed contained 1~3 VgPGCs in the migratory route at earlier stages. Notably, many such chimeras had 2~5 VgPGCs in the gonad, as shown for representatives from class-III chimeras (Fig. 3a,a’) and class-IV chimeras having 7 VgPGCs each (Fig. 3b,b), suggesting proliferation of donor PGCs during and post migration.

### Germline transmission

In order to evaluate germline transmission, donor fish and BCT-derived chimeras were grown into adulthood and tested for their fertility by breeding as described above for the host. Chimeras of classes I and II from BCT into normal hosts exhibited fertility rates of 100% and 98.4% fully comparable to 98.3 for non-transplanted hosts (Table 2). Notably, chimeras of classes III from BCT into dnd-depleted hosts exhibited a reduced fertility rate of 91.4%, which suggests that transplantation of 30 dnd-overexpressing blastula cells containing 2.5 PGCs is able to restore the fertility in more than 90% of chimeras (Table 2). The fertility rate value 91.4% is similar to 92.4% as the germline chimera frequency predicted from the BCT conditions by the binorminal distribution (Table 2). In addition, chimeras of classes IV from BCT into γ-irradiated hosts also exhibited a reduced fertility rate of 92.3%.

Fertility-proven donor fish and chimeras were subjected to progeny test via crossing with *i*^*3*^, and resultant embryos and fry were observed for pigmentation, liver and gonad. Among 8 donor fish, 8, 6 and 5 were found to be transmitters of pigmentation, Vg and Lr. They produced 494 F1 progeny embryos, of which 48.6%, 33.2% and 33.6% were positive for black pigmentation, Vg and Lr (Table 3, Table S2), which are in accordance with 50% for the pigmentation locus and 33.3% for the Vg and Lr transgenes in BGR donor embryos. Doubling of 48.6% and tripling of 33.2% and 33.6% led to genotype-calibrated transmission rates 97.2%, 99.6% and 100.8% for pigmentation, Vg and Lr, producing an average of 99.2% as the genotype-calibrated general germline transmission rate for the donor (Table 3).

Among 6 class-I chimeras progeny-tested, 2, 2 and 1 were found to be transmitters of pigmentation, Vg and Lr. They produced 881 F1 progeny embryos, of which 2.4%, 1.7% and 1% were positive for black pigmentation, Vg and Lr, generating an average of 4.3% as the genotype-calibrated general germline transmission rate for class-I chimeras (Table 3, Table S2). Among 6 class-II chimeras progeny-tested, 6, 4 and 6 were found to be transmitters of pigmentation, Vg and Lr. They produced 380 F1 progeny embryos, of which 13.2%, 6.8% and 15.8% were positive for black pigmentation, Vg and Lr, giving rise to an average of 31.4% as the genotype-calibrated general germline transmission rate for class-II chimeras (Table 3, Table S2). Comparing 31.4% of class-II chimeras with 4.3% of class-I chimeras revealed a 7.3-fold enhancement in germline transmission rate. Taken together, PGC boosting in donor embryos by dnd overexpression is able to greatly increase germline chimera production.

A total of 10 class-III chimeras were progeny-tested. Among them, 10, 9 and 10 were found to be transmitters of pigmentation, Vg and Lr. They produced 609 F1 progeny embryos, of which 49.9%, 30.2% and 33%% were positive for black pigmentation, Vg and Lr. Calibration to the donor genotype led to an average of 96.5% as the general germline transmission rate for class-III chimeras (Table 3, Table S2). This value is comparable to 99.2% of the donor. It deserves to note that one of the 10 chimeras incapable of transmitting Vg was a male capable of transmitting pigmentation at 52.5% and Lr at 40,7% to its F1 progeny (serial number 6, Table S2), demonstrating that its germline is of donor origin. Taken together, germ cells in fertile class-III chimeras are exclusively of donor origin, and the combination between PGC boosting by dnd expression in the donor and PGC ablation by dnd depletion in the host allows for a high efficiency of germline chimera formation and 100% GR in medaka.

### Chimeric liver and gonads of germline chimeras

We examined the chimerism degree in adult organs of progeny-tested chimeric Vg and Lr transmiters of classes III and III. In the ovary of a typical class-II chimera, GFP-positive female germ cells of donor origin coexisted with many GFP-negative counterparts largely of host origin (Fig. 3c). In the testis and ovary of class-III chimeras, the proportion of GFP-positive germ cells was remarkably enhanced (Fig. 3d,e). In the liver of both class-II and -III chimeras, RFP-negative and –positive cells were easily detectable in the whole organ and its squash (Fig. 3f–i). These results together with fertility data, donor genotypes and phenotypes as well as chimeric germline transmission demonstrate that BCT between dnd-overexpression donor and dnd-depleted host is able to achieve complete GR without affecting somatic chimerism.

## Discussion

In this study, we have developed a BCT procedure that allows for the generation of fertile fish invariantly with the germline of completely donor origin. On one hand, we show that PGC-depleted embryos invariantly develop into germ cell-less sterile adults, whereas 91.4% of class-III chimeras from PGC-depleted hosts transplanted with 30 PGC-boosted blastula cells containing 2.5 PGCs develop into fertile females and males. Since the value of 91.4% is indifferent from 92.4% predicted as the frequency of PGC transplantation from the binorminal distribution, the introduction of 1 or few donor PGCs into the majority – if not all - of chimeras is able to restore fertility. On the other hand, there is no difference in germline transmission and phenotypic segregation between chimeras and donor fish, demonstrating the absence of host germline. In summary, the BCT procedure reported in this study is able to restore fertility in PGC-deficient embryos at an extremely high efficiency and ensure that all fertile chimeras generate gametes of donor origin, a crucial factor for restoration and propagation of biodiversity.

Our success is attributed to the combinational use of two approaches. One is 100% PGC elimination in the host by *dnd* depletion. Prevention of PGC formation and interference with PGC migration or survival in subsequent development may compromise and abolish the germline and fertility at varying efficiencies. We have recently reported *dnd* as the PGC specifier in medaka and its depletion prevents PGC formation^1^. PGC elimination by compromising PGC migration or survival has been achieved by *dnd* depletion in several fish species including zebrafish^3^,^18^,^39^,^40^, loach^41^, goldfish^42^, starlet^43^ and Atlantic salmon^44^. This study corroborates and extends these reports by demonstrating the ability of *dnd* depletion for 100% host sterilization during embryonic and adult development.

The other approach to the 100% GR is ascribed to the new addition of boosting PGCs in the donor by *dnd* overexpression. One of major challenges for BCT-mediated GR is that only a small proportion of blastula cells are PGCs or PGC-competent cells capable of germline contribution. In this study we show that dnd overexpression is able to elevate the PGC proportion from ~3.3% to ~8.2%, in accordance with our previous report that *dnd* is not only necessary for PGC specification but also sufficient for increasing PGCs via boosting PGC precursors in medaka^1^. More importantly, the use of PGC-boosted donor blastula cells is indeed able to increase the efficiency of germline chimera production by 7 folds. Identification of factors capable of remarkably increasing the PGC number will be valuable for efficient GR by BCT. It has been reported that overexpression of several germ genes such as *vasa* and *piwi* is generally unable to increase the PGC number in zebrafish^45^ and medaka^22^,^38^. There are two exceptions. One is the zebrafish *buc* whose overexpression is capable of increasing the PGC number by up to ~50% 46. The other is *dnd* in medaka as shown here and previously^1^. The ability of *dnd* to boost PGCs has so far been limited to medaka, as its overexpression in zebrafish does not increase the PGC number^26^.

GCT and BCT feature their respective advantages and are perfectly complementary each other for different applications. GCT is of choice for the propagation of scientifically or commercially valuable but rare or super animals. In this case, embryos are either rare and thus limited in number or longer available (e.g. the embryo that has giving rise to a super animal) for donor blastula cell preparation, whereas biopsies may be taken to enable germ cell preparation and propagation into a large for surrogate production of numerous female and male progeny via GCT. Consequently, surrogate progeny from a single super animal may establish a self-mating population to further increase the individual number by normal sexual reproduction. Since the progeny could originate from a single genotype, the self-mating system will minimize genetic variations arising from meiotic segregation and assortment and thus maximize the probability to reconstitute the genotype and phenotype of a super animal.

BCT is, on the other hand, of choice particularly for biodiversity conservation via restoration and propagation of endangered species. There are three key factors essential for surrogate production of biodiversity, namely donor-host immunocompatibility, purity and diversity of progeny animals. BCT perfectly fulfil these requirements. There is no immune rejection in developing fish embryos as has been well-demonstrated by the production of intra-species chimeras^17^,^18^,^21^,^35^,^37^,^47^ and even interordinal chimeras^48^. Easy and reliable PGC ablation by *dnd* depletion ensures GR-dependent fertility restoration and thus a pure donor origin of progeny from BCT chimeras. As for diversity, donor blastula cells may be collected from hundreds and thousands of PGC-boosted blastulae and thus rich in genetic diversity. BCT may introduce PGCs from different donor embryos into the chimeric germline. Consequently, PGCs in different BCT chimeras and even one and the same chimera may differ in individuality and thus genotype, which makes an apparent sense in terms of effective population size (N_e_), a parameter essential for biodiversity conservation and management. In practice, a N_e_ of 100 is considered as the minimal value necessary for biodiversity restoration and maintenance. If a population has 50 males and 50 females, then N_e_ is 100 [(4 × 50 × 50)/(50 + 50)] according to the equation N_e_ = (4N_m_N_f_)/(N_m_ + N_f_), where N_e_ is the effective population size; N_m_ is the number of males; and N_f_ is the number of females. In this study we have shown that medaka BCT chimeras often have ~2.5 PGCs of different origins and thus genotypes. Consequently, ~20 male and ~20 female BCT chimeras will approach this minimal N_e_ value. Blastula cells are easy for dissociation and testing for viability by observation of pseudopodial formation and cell division and do not need to characterize the cell identity as they either contain or produce PGCs in diverse animals^1^,^4^,^5^,^6^. Blastula hosts are also easy for preparation, cell transplantation and judgement of successful BCT via monitoring donor cells’ behaviors during subsequent development^34^,^35^,^49^. BCT is particularly ideal for surrogate production of animals that have high fecundity and robust embryology, a feature common to many lower vertebrates such as frogs and fish. In addition, BCT is the only strategy to preserve and propagate scientifically valuable mutants capable of blastula formation but die from abnormal subsequent development or incapable of normal gametogenesis in a somatically defect gonad, as has been illustrated in zebrafish^18^.

Several fundamental questions remain to be answered before surrogate production can be used in conservation practice. These include donor-host phylogeny and developmental-physiological compatibility in gametogenesis and fertilization. Our finding that BCT can achieve a high efficiency of fertility restoration and GR makes medaka an ideal model organism for the experimental analysis of these biological parameters towards BCT-mediated surrogate production and propagation of endangered lower vertebrates and biodiversity.

## Methods

### Chemicals and fish

Chemicals were purchased from Sigma, enzymes were from New England Biolabs, and PCR reagents were from TaKaRa unless otherwise indicated. Fish work was performed in strict compliance with the recommendations in the Guide for the Care and Use of Laboratory Animals of the National Advisory Committee for Laboratory Animal Research in Singapore and approved by this committee (Permit Number: 27/09). Medaka strains HB32C, *i*^*3*^, HdrR and transgenic lines (see below) were maintained under an artificial photoperiod of 14-h light to 10-h darkness at 26 °C as described^34^.

### Morpholino oligo and plasmids

MOdnd and pCSdnd:chDD were described^1^, the former is morpholino antisense oligo targeting the medaka dnd mRNA around the translational initiation, the latter expresses a fusion between the medaka Dnd protein and cherry protein. pLFABP-rfp contains the 2.8-kb liver-specific promoter of the zebrafish liver fatty acid binding protein and drives RFP expression specifically in embryonic and adult liver in zebrafish^50^ and medaka^34^.

### Microinjection

Medaka embryos were injected at the 1-cell stage with 20~50 pg of MOdnd or 100 pg of capped dnd:ch mRNA synthesized from pCSdnd:chDD as described^1^,^22^,^51^.

### Cell transplantation

Preparation of donor blastula cells, dechorionated host MBEs and cell transplantation were performed essentially as described^21^,^22^,^34^. MBEs of albino strain *i*^*3*^ were used as the transplantation host. The transplantation donor was generated in multiple steps as follows. HB32C is a wild-type pigmentation strain^52^. Lr is a liver-red transgenic line of strain orange produced by microinjecting pLFABP-rfp. Vg is a transgenic line of see-through medaka Wakamatsu *et al*., 2001 that expresses GFP from the medaka Vasa promoter specifically in germ cells^22^. Crossing between Lr and Vg led to F1 fish that expresses GFP in germ cells and RFP in the liver on the *i*^*3*^ albino background. F1 fish positive for Vg and Lr were mated to produce F2 generation. F2 embryos positive for Vg and Lr were grown to adults designated as GL fish that were homozygous (1/3) or heterozygous (2/3) for Vg and Lr transgenes and thus 66.6% for Vg and Lr phenotypes. Finally, GL females were crossed with HB32C male, generating BGL embryos that were used as the transplantation donor. BGR is heterozygous (50%) to black melanophores responsible for wild-type pigmentation and 33.3% for Vg and Lr transgenes and phenotypes. In some experiments, donor embryos at the 1-cell stage were injected with 100 pg of dnd:ch RNA to increase the PGC number, and host embryos also at the 1-cell stage were injected with 20–100 ng of MOdnd to ablate PGCs or at the 4- to 8-cell stages were subjected to γ-irradiation at 6 gy^34^.

### Fertility test

Adult fish from MOdnd-injected *i*^*3*^ embryos were first maintained on their own for 1 month to observe egg production. They were then maintained for 2 weeks in the presence of fertile HdrR males and another 2 weeks in the presence of fertile HdrR females. Adult chimeras were examined similarly by using fertile HdrR males and fertile HdrR females as the testers. Fertile females were proven by egg-laying. Fertile males were proven by the ability to fertilize eggs in pair-wise mating.

### Germline transmission

Fertile male and female chimeras were individually test-crossed with *i*^*3*^ albino medaka. Progeny were phenotypically analyzed for the donor contribution by pigmentation, GFP and RFP expression.

### Histology of chimeras

The liver, ovary and testis of progeny-tested chimeras were dissected and examined for donor cell derivatives by GFP and RFP expression.

### Microscopy

Observation and photography on Leica MZFIII stereo microscope, Zeiss Axiovertinvert and Axiovert upright microscopes with a Zeiss AxioCam M5Rc digital camera (Zeiss Corp) were as described previously^21^,^28^,^34^.

### Statistics

Statistical analyses were calculated by using Graphad Prism v4.0. Data consolidated were presented as mean ± s.d. and p values were calculated by using non-parametric student’s *t*-test. Genetic data were evaluated by using *chi*-square test.

## Additional Information

**How to cite this article**: Li, M. *et al*. Germline replacement by blastula cell transplantation in the fish medaka. *Sci. Rep.* **6**, 29658; doi: 10.1038/srep29658 (2016).

## Supplementary Material

Supplementary Information

## Figures and Tables

**Table 1:** Phenotypes of donor and chimeric embryos^1^.

Sample		Embryos observed	Pigmented, n (%)	Lr-positive, n (%)^2^	Vg-positive PGC		Total PGCs predicted^4^	
					n (%)^2^	PGC^3^	%	PGC^3^
BGR donor	normal	31	31 (100)	20 (64.5)	21 (67.7)	33.1 ± 2.93	100	33.1 ± 2.93
	dnd RNA	30	30 (100)	20 (66,6)	20 (66.7)	82.5 ± 5.42	100	82.5 ± 5.42
Chimera^5^	Class I	36	36 (100)	21 (58.3)	17 (47.2)	1.41 ± 0.51	63.5	1.6 ± 0.8
	Class II	32	32 (100)	18 (56.2)	26 (81.2)	2.08 ± 0.74	92.4	2.7 ± 1.3
	Class III	37	37 (100)	21 (56.8)	30 (81.1)	2.07 ± 0.78		
	Class IV	24	24 (100)	18 (75.0)	19 (79.2)	2.21 ± 0.71		

^1^Vg was observed at stages 18–23 when PGCs were positioned bilaterally to somites and easily countable. Melanocyte and Lr were observed from 5 dpf onwards. All embryos are positive for pigmentation.

^2^Comparisons between embryos observed and positive for Lr or Vg.

^3^Number of heterozygous VgPGCs per embryos presented as means ± sd.

^4^Percentages of donor PGC-containing embryos and numbers of total PGCs. These values in donor embryos are the same as experimentally determined, and in chimeras were predicted from the genotype of BGR embryos (see Fig. 1) and the bimodal distribution by using 1 PGC including 0.66 VgPGC [2/3(33.1 PGCs) per 1000-cell donor blastula; class I) and 2.5 PGCs including 1.65 VgPGCs [2/3(82.5 PGCs) per 1000-cell donor blastula; classes II-IV] as the input numbers of PGCs within 30 donor blastula cells transplanted.

^5^For chimera classes see Fig. 2.

**Table 2:** Survival and fertility of hosts and chimeras^1^.

Type	Treatment	Embryo sampled	Adult, n (%)^2^	Fertile, n (%)^3^
i^3^ host	Control	132	59 (44.7)	58 (98.3)
	γ-irradiation (6 gy)	379	37 (9.8)	35 (94.6)
	MOdnd (20 pg)	73	31 (42.5)	10 (32.2)
	MOdnd (50 pg)	121	49 (40.5)	0 (0)
	MOdnd (100 pg)	226	98 (43.4)	0 (0)
Chimera^4^	Class I	156	56 (35.9)	56 (100)
	Class II	173	61 (35.2)	60 (98.4)
	Class III	188	58 (32.2)	53 (91.4)
	Class IV	154	13 (8.4)	12 (92.3)

^1^Fish from embryos with or without BCT were maintained for testing fertility, the ability to produce F1 progeny embryos, from 3 months post hatching onwards by massive breeding. Fertility was judged by egg production for females and egg-fertilizing ability for males. Massive breeding was performed first on experimental fish for one month, followed by 2 weeks of breeding with added fertility-proven HdrR males and another 2 weeks of breeding with added fertility-proven HdrR females. Fertile fish were randomly chosen for progeny test (see Table 3).

^2^This is the survival rate by comparison between embryos samples and adults obtained.

^3^This is the fertility rate by comparison between total adults obtained and fertile adults.

^4^Chimeras produced by transplanting ~30 blastula cells into each blastula host. Classes I-IV are BCT between normal donor and host (class I), dnd RNA-injected (100 pg) donor and normal (class II), MOdnd-injected (100 pg; class III) and γ-γ-irradiated hosts (class IV). For more details see Fig. 2.

**Table 3:**
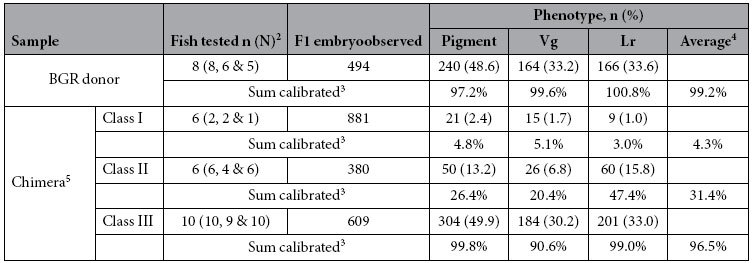
Germline transmission of donor and chimeras^1^.

^1^Fertile male and female after fertility test (see Table 1) were randomly chosen for test cross with albino host *i*^*3*^ of opposite sexes, and F1 embryos were examined for Vg, Lr and pigmentation phenotypes.

^2^n, total number of fish individuals tested; N, numbers of germline transmiters for pigment, Vg and Lr.

^3^Percentage germline transmission was calibrated to 50% (heterozygosity) for pigment and to 1/3 for Vg and Lr by doubling the observed percentage values for pigment and tripling the observed percentage values for Vg and Lr, respectively.

^4^Average of calibrated percentage values for pigment, Vg and Lr.

^5^For chimera classes see Fig. 2 and Table 1.
