# Supplementary material for: Germline replacement by blastula cell transplantation in the fish medaka
Source: Sci Rep. 2016 Jul 13;6:29658. doi: 10.1038/srep29658 (PMC4942801; doi:10.1038/srep29658)
Supplement: Supplementary Information [file srep29658-s1.doc]

**Germline replacement by blastula cell transplantation in the fish medaka**

Mingyou Li, Ni Hong, Hongyan Xu, Jianxing Song, Yunhan Hong

**Table S1 | Phenotypes of donor and chimeric embryos 1)**

| Serial | HGR | | Chimera | | | |
| --- | --- | --- | --- | --- | --- | --- |
|  | normal | Dnd RNA | Class I | Class II | Class III | Class IV |
| 1 | 35 | 79 | 2 | 2 | 2 | 2 |
| 2 | 32 | 86 | 2 | 2 | 2 | 2 |
| 3 | 29 | 91 | 1 | 2 | 1 | 3 |
| 4 | 25 | 73 | 1 | 1 | 3 | 1 |
| 5 | 37 | 68 | 2 | 3 | 4 | 2 |
| 6 | 33 | 83 | 1 | 4 | 1 | 2 |
| 7 | 32 | 85 | 2 | 2 | 2 | 4 |
| 8 | 36 | 89 | 1 | 1 | 3 | 2 |
| 9 | 31 | 87 | 1 | 2 | 2 | 2 |
| 10 | 34 | 81 | 2 | 3 | 1 | 3 |
| 11 | 33 | 80 | 1 | 2 | 3 | 3 |
| 12 | 31 | 85 | 1 | 2 | 2 | 2 |
| 13 | 35 | 78 | 2 | 2 | 2 | 1 |
| 14 | 37 | 79 | 1 | 2 | 2 | 2 |
| 15 | 34 | 84 | 1 | 3 | 2 | 3 |
| 16 | 33 | 82 | 1 | 1 | 1 | 2 |
| 17 | 38 | 88 | 2 | 3 | 3 | 2 |
| 18 | 33 | 83 |  | 2 | 2 | 2 |
| 19 | 32 | 86 |  | 2 | 2 | 2 |
| 20 | 31 | 82 |  | 1 | 3 |  |
| 21 | 34 |  |  | 2 | 1 |  |
| 22 |  |  |  | 1 | 2 |  |
| 23 |  |  |  | 2 | 2 |  |
| 24 |  |  |  | 3 | 3 |  |
| 25 |  |  |  | 2 | 1 |  |
| 26 |  |  |  | 2 | 3 |  |
| 27 |  |  |  |  | 2 |  |
| 28 |  |  |  |  | 1 |  |
| 29 |  |  |  |  | 2 |  |
| 30 |  |  |  |  | 2 |  |

^1)^Vg was observed at stages 18-23 when PGCs were positioned bilaterally to somites and easily countable.

**Table S2 | Germline transmission of donor and chimera individuals ^1)^**

| Class | Donor | Host | Fish | F1, n | Phenotypes, n (%) | | |
| --- | --- | --- | --- | --- | --- | --- | --- |
|  |  |  |  |  | Melanocyte | Vg | Lr |
| HGR donor control | | | 1 ♂ | 98 | 46 | 0 | 48 (49.0) |
|  |  |  | 2 ♂ | 64 | 31 | 30 (46.9) | 33 (51.6) |
|  |  |  | 3 ♂ | 73 | 36 | 34 (46.6) | 38 (52.1) |
|  |  |  | 4 ♂ | 55 | 29 | 26 (47.3) | 0 |
|  |  |  | 5 ♀ | 46 | 22 | 21 (45.7) | 22 (47.8) |
|  |  |  | 6 ♀ | 61 | 30 | 32 (52.5) | 0 |
|  |  |  | 7 ♀ | 53 | 25 | 0 | 25 (47.2) |
|  |  |  | 8 ♀ | 44 | 21 | 21 (47.7) | 0 |
|  |  |  | sum | 494 | 240 (48.6) | 164 (33.2) | 166 (33.6) |
| Class I  chimera | normal | normal | 1 ♂ | 243 | 0 | 0 | 0 |
|  |  |  | 2 ♂ | 258 | 18 (7.0) | 13 (5.0) | 0 |
|  |  |  | 3 ♂ | 186 | 0 | 0 | 0 |
|  |  |  | 4 ♀ | 96 | 3 (3.1) | 2 (2.1) | 9 (9.4) |
|  |  |  | 5 ♀ | 44 | 0 | 0 | 0 |
|  |  |  | 6 ♀ | 54 | 0 | 0 | 0 |
|  |  |  | sum | 881 | 21 (2.4) | 15 (1.7) | 9 (1.0) |
| Class II  chimera | Dnd RNA | normal | 1 ♂ | 64 | 4 (6.3) | 4 (6.3) | 2 (3.1) |
|  |  |  | 2 ♂ | 85 | 12 (14.1) | 6 (7.1) | 9 (10.6) |
|  |  |  | 3 ♂ | 60 | 17 (28.3) | 14 (17.2) | 21 (35.0) |
|  |  |  | 4 ♀ | 37 | 5 (13.5) | 0 (0) | 12 (32.4) |
|  |  |  | 5 ♀ | 68 | 5 (7.4) | 2 (2.9) | 9 (13.2) |
|  |  |  | 6 ♀ | 66 | 7 (11.7) | 0 (0) | 7 (10.6) |
|  |  |  | sum | 380 | 50 (13.2) | 26 (6.8) | 60 (15.8) |
| Class III  chimeras | Dnd RNA | MOdnd | 1 ♂ | 78 | 36 (46.2) | 17 (21.8) | 25 (32.1) |
|  |  |  | 2 ♂ | 65 | 30 (46.2) | 26 (40.0) | 18 (27.7) |
|  |  |  | 3 ♂ | 80 | 35 (43.8) | 20 (25.0) | 10 (12.5) |
|  |  |  | 4 ♂ | 66 | 30 (45.5) | 20 (30.3) | 23 (34.8) |
|  |  |  | 5 ♂ | 52 | 34 (65.4) | 32 (61.5) | 27 (51.9) |
|  |  |  | 6 ♂ | 59 | 31 (52.5) | 0 (0) | 24 (40.7) |
|  |  |  | 7 ♀ | 39 | 21 (53.8) | 11 (28.2) | 23 (59.0) |
|  |  |  | 8 ♀ | 70 | 39 (55.7) | 22 (31.4) | 17 (24.3) |
|  |  |  | 9 ♀ | 67 | 30 (44.8) | 25 (37.3) | 24 (35.8) |
|  |  |  | 10♀ | 33 | 18 (54.5) | 11 (33.3) | 10 (30.3) |
|  |  |  | sum | 609 | 304 (49.9) | 184 (30.2) | 201 (33.0) |

**^1)^**Pooled BGR embryos at the 1-cell stage were not injected or injected with *dnd;ch* RNA (100 pg/embryo), dissociated at the midblastula stage into single cells for transplantation into non-transgenic *i^3^* blastula hosts with or without injection of MOdnd (1 ng/embryo) at the 1-cell stage. Fertile chimeras were examined for germline transmission by using non-transgenic albino *i^3^* for test crosses. F1 embryos and fry were observed for Lr, Vg and melanocytes.
